# Insights on Public Health Professionals Non-technical Skills in an Emergency Response (Multi-Team System) Environment

**DOI:** 10.3389/fpsyg.2022.827367

**Published:** 2022-06-14

**Authors:** Andrew Black, Olivia Brown, Heini Utunen, Gaya Gamhewage, Julie Gore

**Affiliations:** ^1^Health Emergencies, Learning and Capacity Development Unit, World Health Organization, Geneva, Switzerland; ^2^School of Management, University of Bath, Somerset, United Kingdom; ^3^Department of Organizational Psychology, Birkbeck College, University of London, London, United Kingdom

**Keywords:** multiteam systems, non-technical skills, public health professionals, emergency-response, critical decision method

## Abstract

This paper provides practitioner and academic insights into the importance of examining non-technical skills in a multiteam system emergency response. The case of public health professionals is highlighted, illustrated with unique qualitative field data which focused upon the use of non-technical skills at a meso level of analysis. Results reflected the importance of context upon the multiteam system and highlighted seven non-technical skills used by public health professionals to support an effective response. Recommendations for future research and implications for practice are noted for this hard to access professional group, located within emerging advances in the scientific inquiry of complex and increasingly evident, multi-team systems.

## Introduction

The number of disasters and outbreaks to which national governments and the international community respond has increased in frequency in the 21st century (World Health Organization, 2017a). Responding to such incidents is becoming more challenging, as communities become increasingly urbanized and the world more interconnected, disease outbreaks that were previously localized can now travel across borders and spread globally within days. The COVID-19 pandemic offers an extreme example of the way in which a health threat can quickly spread across the world, impacting not only public health, but economies, politics and society (Independent Panel for Pandemic Preparedness and Response, 2021; Pereznieto and Oehler, 2021).

To address complex emergencies, governments have developed emergency management systems that bring together people with a range of technical expertise, from a number of different agencies to prepare for and direct the response to emergencies (Australian Institute for Disaster Resilience, 1998; Cabinet Office, 2012; JESIP, 2013; FEMA, 2017; Luciano et al., 2018). At a national and international level, the International Health Regulations outline the capacities governments should develop within these emergency management systems, to manage the health impacts of emergencies, including not only the detection and management of health consequences but the requirement to limit spread and impact on the economy (World Health Organization, 2017a,b,c).

In the academic literature, these complex networks of teams, formed within the emergency management framework are referred to as multi-team systems (MTSs)—a network of component teams working to achieve separate objectives within the framework of collective, over-arching shared goals (Mathieu et al., 2001). Due to their diverse and multi-faceted nature, MTSs are especially suited to responding in complex, high-stakes emergencies, however, the coming together of multiple agencies can create difficulties in collaboration and coordinated action (Marks et al., 2005; Fodor and Flestea, 2016; Brown et al., 2021).

Reviews of major emergencies, over the last 20 years have argued that improving the use of non-technical skills can enable responders to overcome some of these challenges (Pollock, 2017; Kerslake Report, 2018). These observations have been met with increased research into non-technical skills in emergency response environments. For instance, research has examined skills such as communication and coordination in military personnel, the blue-light emergency services and search and rescue teams (Fodor and Flestea, 2016; Wijnmaalen et al., 2019; Brown et al., 2021). There remains however, an absence of research examining the non-technical skills used by public health responders, in addition to limited empirical studies of non-technical skills in complex MTSs. This study begins to address this research gap, by qualitatively identifying the non-technical skills used by public health responders in a complex multi-team environment.

## Public Health Responders – A Multi-Team Environment

Studies of MTSs have shown that traditional teamwork models cannot explain the operational reality of the response environment where multiple teams work together (Lanaj et al., 2013; Zaccaro et al., 2012; Luciano et al., 2018). While most conventional teams operate with a degree of stability, MTSs are characterized by dynamism and fluidity (Luciano et al., 2018). This is recognizant of a public health response, in which complex networks of teams are formed *ad hoc*, comprised of individuals from different disciplines, different organizations, brought together to resolve particular issues (Miller et al., 2008; Khan et al., 2018; Soujaa et al., 2021). Coordination of responses involving multiple stakeholders is difficult due to having multiple parallel response systems operating simultaneously: governmental response systems, humanitarian organizations and communities competing for scarce resources (Lanzara, 1983; Hicks and Pappas, 2006; Global Public Policy Institute, 2010; ALNAP, 2012; Junger, 2016). This complexity increases pressure on response staff who are often called to work with more groups than usual, many of which will be unfamiliar (CARE, 2005; Global Public Policy Institute, 2010; United Nations Development Programme, 2016); operate to different standards (Owen and Hayes, 2014); and work under stress (McLennan et al., 2014) in rapidly changing environments to which they must adapt (Comfort and Kapacu, 2006; Rees-Gildea and Moles, 2012).

A further challenge facing public health responders is the need to balance the aims and priorities of multiple different agencies. This is reflected upon in the literature on MTSs, which outlines how component teams must respond effectively to their own organizational priorities (e.g., healthcare professionals must provide care and treatment to members of the public), while simultaneously remaining mindful of the wider over-arching priorities of the multi-team network (e.g., to limit the spread of disease and minimize the impact on the economy) (see Marks et al., 2001; Luciano et al., 2018; Rico et al., 2018; Ward et al., 2020). Managing this complex hierarchy of priorities, alongside the need to maintain effective communication with the other teams within the MTS network is challenging and can lead to disagreements over whose objectives should take priority (Waring et al., 2020; Brown et al., 2021).

To date, much of the research on MTSs has been theoretical, seeking to establish structural frameworks to outline how these complex networks of teams operate (see Zaccaro et al., 2012; Luciano et al., 2018; Shuffler and Carter, 2018). Similarly, in the public health context, organizations have focused on the structural specifics of emergency management systems (as opposed to the people operating within those system) when seeking to address prior failures in response—planning, training and after-action reviews have concentrated on bolstering systems to try and eradicate the possibility of human error, systemic lessons, high-level leadership, or blanket calls for staff with “more experience” (CARE, 2005; Global Public Policy Institute, 2010; Rees-Gildea and Moles, 2012; United Nations Development Programme, 2016). For example, lessons from the 2010 Haiti earthquake and subsequent cholera outbreak prompted reforms to the UN Cluster response system (World Health Organization, 2020); the 2014–15 West Africa Ebola outbreak prompted changes to WHO’s response structure (Gostin and Friedman, 2015; Heymann et al., 2015; Moon et al., 2015; World Health Organization, 2015).

Notably, academics and practitioners are increasingly paying attention to the skills and training needed by people to ensure that they can work effectively (and adaptively) within these systems, due to a series of failures that have been attributed to difficulties at the human level (Hollnagel and Woods, 2005; Reason et al., 2006). Indeed, studies of large-scale international and domestic responses such as: the 2010 Haiti Earthquake and 2005 Tsunami response (ALNAP, 2013); the 9/11 attacks, Hurricane Katrina and the French 2004 heatwave point to challenges in leadership, decision-making, communication, and coordination between multiple agencies (Comfort et al., 2004; Lagadec, 2004; Comfort and Kapacu, 2006; Waugh and Streib, 2006; Comfort, 2007; Farazmand, 2007; Knox-Clarke, 2013).

## The Importance of Non-Technical Skills

Cognitive science challenges the assumption that the design of increasingly complex systems that address all eventualities, provides the answer to system failures (Reader and O’Connor, 2014; Flin et al., 2017). Systems are designed to address complete and well-defined problems whereas humans are creative, flexible and can adapt to complex environments (Bram and Vestergren, 2012). People, unlike systems, can rapidly compensate for changes in the environment that impact system performance and compensate for lack of information and uncertainty to make decisions with incomplete information (Elbright et al., 2003). In complex emergency environments technical knowledge and systems alone are not enough. Response systems must allow for the interaction between static procedures and a human-being’s capacity to learn, innovate and adapt to changing circumstances (Comfort, 2007; Global Public Policy Institute, 2010; ALNAP, 2012).

A necessary alternative to solely focusing on the structure of the emergency MTS, is a focus on the skills required by humans to work effectively within the system—non-technical skills. Non-technical skills are “cognitive, social and personal resource skills that complement technical skills and contribute to safe and efficient task performance,” that underpin safe performance in high-risk, extreme contexts (p.1, Flin et al., 2008; Reader and O’Connor, 2014). The study of non-technical skills is principally concerned with helping individuals and teams overcome challenges when systems fail and has been identified as a means of improving both safety and efficiency of teamwork in a range of contexts including aviation, firefighting, the military and health (Weick, 1993; Flin et al., 2008; Owen, 2014; see Table 1 for an overview).

**TABLE 1:** Core non-technical skills with a brief explanation (adapted from Flin et al., 2008).

Skill	Elements
Situation Awareness	Gathering and interpreting information, anticipating future states. Situational awareness is the ability to picture and assess a situation. It plays a major part in decision-making. A lack of situational awareness can lead to staff fixating on relatively minor problems and failing to acknowledge larger dangers or failing to identify the most important problems to be addressed.
Decision-making	Defining a problem, considering and selecting options: In the context of emergencies decision-making requires reaching a judgment about the situation, choosing a course of action (often rapidly and with limited information) and then reviewing that decision as part of an on-going process
Communication	Sending, receiving and contextualizing information. Poor communication has often been cited as a cause of accidents. It can be shaped by policy (for example the use of jargon) but also requires staff to not only send but to receive information appropriately.
Team working	Supporting and coordinating. A key factor is about making individuals more effective in the teams in which they are working. This focuses on how team members define tasks and roles in order to work more effectively
Leadership	Planning, use of authority, maintenance of standards and discipline. Effective planning and coordination within a team and with other teams is a key element of the response.
Managing Stress	Identifying causes of both chronic and acute stress, recognizing the symptoms and effects and implementing coping strategies
Coping with fatigue	Identifying the causes of fatigue, recognizing the effects of fatigue and implementing coping mechanisms

Non-technical skills like decision-making, leadership, communication, and coordination have been identified as key components to enable the successful management of health emergencies and to enable team members to operate effectively complex, multi-agency networks (Prineas et al., 2021). However, despite several reports highlighting the importance of technical skills in crises there have been no empirical studies of non-technical skills used by public health staff working in emergencies (Global Public Policy Institute, 2010; ALNAP, 2012). Furthermore, non-technical skills have predominantly been examined in individual teams and not in multi-team systems that characterize most emergency responses (see Flin et al., 2008). Establishing an empirical evidence base to illustrate how NTSs might be used in MTSs is therefore important, especially due to the challenges of applying findings from conventional teams to teams operating within a multi-team context (see Zaccaro et al., 2012; Lanaj et al., 2013).

In the current study, we aimed to qualitatively explore the experiences of public health professional’s response to disease outbreaks. Set within this theoretically challenging and somewhat limited evidence base, gaining access to this hard to access professional group warranted more detailed attention. Drawing on the gaps in extant literature, the current research therefore had two aims: (i) to characterize Public Health professional’s response to emergencies as a multi-team system environment and (ii) to identify the non-technical used by responders to support effective response.

## Method

### Participants

Once ethical approval was obtained, participants were invited to take part in the research *via* email. A purposive sampling approach was adopted to identify 10 public health professionals, with a minimum of 10 years-experience and who had held the most commonly deployed roles utilized in the Global Outbreak Alert and Response Network (MacKenzie et al., 2014). The roles include—coordinators, laboratory researchers, risk communication and community engagement practitioners, infection prevention control specialists and epidemiologists.

### Procedure:

### Data Collection

All participants were interviewed using the Critical Decision Method, (see Table 2, Interview protocol) a retrospective, cognitive task analysis, narrative-based interviewing technique, designed to use probe questions to gather information and analyze non-routine events which require the application of judgment in complex environments with small numbers of expert participants (interviewing 5–6 experts usually reaches data saturation *see* Klein et al., 1989; Boulton and Cole, 2016; Gore et al., 2017; Power and Alison, 2017; Militello and Anders, 2020; Brown et al., 2022). Participants reflected primarily on their experiences responding to Ebola, Cox’s Bazaar and Cyclone Idai outbreaks between 2014 and 2019.

**TABLE 2:** Interview protocol (adapted from Stanton et al., 2013: 96).

Interview stage	Brief description
Incident identification	Participants were asked to identify a challenging incident and one in which they worked alongside additional agencies to their own
Free recall	Participants were offered the opportunity to provide a free recall of the incident without interruption by the interviewer
Timeline verification	The interviewer worked with the participants to construct a timeline of events during the incident
Decision identification	The interviewer worked with participants to identify key decision points during the interview
Probing questions	Probing questions were then utilized to gather further information about the context surrounding the decisions made (adapted from Stanton et al., 2013). For example: - How did you gather the information required to make a decision? - How did you prioritize information provided? - What influenced your decision (e.g., presence or priorities of other agencies)
Additional probing questions	Additional probing questions were included to enable the participants to freely reflect on non-technical skills not already referenced to in their previous answers. For example: - How did you manage experiences of stress during the response? - How did you involve other members of your team? - How did you coordinate members of your team?

The interview comprised five stages, consistent with existing recommendations for conducting CDM and included a further sixth stage to ascertain any additional information about non-technical skills that was not gathered in the initial five stages (Klein et al., 1989; Flin et al., 2008). While the CDM approach tends to focus primarily on decision-making (Crandall et al., 2006; Stanton et al., 2013), in the current study, conversations around challenging decisions were utilized to frame and encourage discussions of other non- technical skills. For example—in asking how participants gathered the information to make a decision, we were interested in capturing how that individual communicated with their inter-team partners. Furthermore—when asking how they dealt with disagreement in their decision, we were interested in capturing how participants managed to maintain collaborative working and align priorities across inter-agency partners. As such, while the interviews did focus in on the decision-making process itself (see results below), decisions taken by responders were also used as a context with which to explore other non-technical skills (e.g., sensemaking, coordination, communication) utilized in the health emergencies and helped to frame conversations around inter-team working. Interviews were 45–90 min in length, with an average length of 65 min.

### Data Analysis

Interviews were transcribed verbatim and analyzed in *Nvivo12* using thematic analysis, a recommended approach for analyzing data obtained through the CDM (Braun and Clarke, 2006; Crandall et al., 2006; Nowell et al., 2017; Power and Alison, 2017). An exploratory thematic analysis was conducted due to the limited research examining non-technical skills in multi-team environments. Data were analyzed using Braun and Clarke’s (2006) six-phased method and combined inductive and deductive analysis (Fereday and Muir-Cochrane, 2006). Following familiarization with the data, deductive coding took place, with the first author drawing on the findings of Flin et al. (2008) to ascertain if the NTSs identified in a surgical context could be found in a MTS context. Next, open coding took place, to identify further NTSs used by the public health professionals to support an effective emergency response. Where appropriate, codes were then collated into high order themes, representative of the dataset of the whole.

## Results

The qualitative extracts from the CDM interview highlight a number of important areas including: characterizing the multi-team environment and identifying non-technical skills.

### Characterizing the Multi-Team Environment

The results clearly indicated that the emergency response environments in which public health staff were working could be examined as MTS. Participants described being an integral part of a wider response environment, involving numerous stakeholders and international partners.

*“… all these actors and these power dynamics and these things, starting from the internal ones and then all these actors and government, you know, and, uh, UN agencies and other NGOs and other local civil society communities, different, uh, political parties, ethnic groups … and then you have to … understand how you can navigate that and be able to operate.”*

They also described themselves as operating within a dynamic and fast-paced environment, in which teams would join and leave the network dependent on the demands in the environment.

*“This is an evolving situation which necessitates many, many decisions all with consequence and careful consideration. They build upon each other. Sometimes I’ve made decisions myself and other times I’ve asked for consultation from the group or other stakeholder.”*

### Identifying Non-technical Skills

**FIGURE 1:**
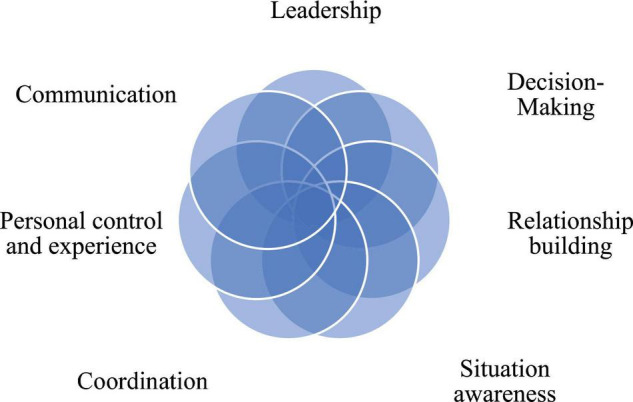
Public health professionals reflections on emergency response: non-technical skill needed to support effective multi team working.

Seven core themes were identified in the data and represent the non-technical skills utilised by the public health responders (see Figure 1 and Table 3). These skills included –*situation awareness, communication, coordination, personal control and experience, relationship building, leadership* and *decision-making*. Four of these skills were identified inductively, drawing on the work of Flin et al. (2008)—*situation awareness, communication, leadership* and *decision-making*. The remaining skills of *coordination, personal control/experience* and *relationship building*, while sharing some overlap with skills identified by Flin et al. (2008) (i.e., team-work and managing stress respectively), were coded for deductively. Several themes comprised sub-themes that were collapsed into high-order core themes due to their overlapping nature. For instance, “*understanding roles and responsibilities*” and “*collaboration*” were collapsed into the core theme of “*coordination.*”

**TABLE 3:** Core themes representing the non-technical skills.

Theme	Sub-themes	Definition
Communication	Briefing	Concise and accurate verbal or written passing of information—briefing is one-way information sharing (from the briefer to the audience)
	Negotiation	Discussion between two or more parties to achieve a common goal—negotiation was required to overcome barriers to cooperation. This was normally described in relation to the decision-making process.
	Ability to provide clear information	The ability to share technical information. This includes orally or by creating technical guidance and advice in the form or using body language
Relationship Building	Developing trust	Building and maintain trust between parties to be able to work effectively together
	Establishing inter-organizational connections	Relationships that are created based on organizational capability and the delivery of work objectives. working together in multi-sector teams helped people to bond, teams came together to share resources, including people and having access to resources seemed in some cases to be a way to shape these relationships
	Developing networks	Identifying and gaining access to people and organizations can help participants complete their task
	Informal communication channels	Creating opportunities to speak across boundaries and hierarchies, this can include organizations or individuals, a network would be with multiple people a channel with an individual perhaps an influencer
Coordination	Understanding roles and capabilities	Understanding the role of the other team(s) in the response and what they contribute
	Collaboration	Recognizing the need for multiple skill sets/functions and teams to build situation awareness and enact decisions—the recognition of needing to work together and the ability to identify which teams can deliver which function. Collaboration requires knowledge of the other partner’s needs, capability and capacity and the needs of the response and the ability to agree and fulfill agreed ways of working
Decision-Making	Joint Decision-making	This is when two or more people arrive at a decision that is to the satisfaction of the group. The solution to a problem or a proposed action was provided by the participant and then debated by the group who either agreed or found a middle way between viewpoints to arrive at a suitable decision. Linked to negotiation there are some instances where joint decision-making was arrived at by brainstorming a joint solution and where following brainstorming the decision-maker overturned the decision of the group
Leadership	Risk Management	Assessing and managing risk; risk was assessed against viable options, prior and technical experience and knowledge, reputation, what was considered “normally done” especially when established processes were not followed.
	Initiative	Taking the lead in decision-making or enacting a decision without consulting others the action is pro-active as opposed to reactive
	Resource Management	Factoring the availability of resources or funding affects the into the decision-making process or way of working
	Providing support	Providing physical support such as working with other teams to achieve an outcome (for example by regular sharing of information and resources) or psychological support by providing advice or encouragement or thanks
Personal control and experience	Resilience	This can be mental and physical. It is demonstrated in the way people react to the environment. It is the ability to detach oneself from emotions and physical difficulty (tiredness for example) to focus objectively on current tasks. This category links to many under self-control which seem to comprise coping mechanisms—the sense of resilience is perhaps at the center of the concept of self-control
	Self-awareness	Knowing and being able to describe your own limits and capabilities both emotional and technical. Being able to describe your own skills, experience and technical knowledge that might build into self-confidence and be part of self-awareness
	Humility	To be able to listen without judgment to the needs and opinions of others and include these into your own reasoning and decision-making
	Pragmatism	Balancing one’s own beliefs and principles with the practicalities of achieving objectives
Situation awareness	Sense-making	Covers information gathering, analysis of the information and predicting future states to develop a coherent picture of what is happening in the response at a given point in time
	Aware of the capacity of others	Being aware of the resources and skills available in your team or organization and in other teams and organizations and how these contribute to the achievement of the mission and distal goal

#### Communication

A key non-technical skill identified was communication. Communication was supported by the ability of responders to brief other members of the response network; persuasion and argument; negotiation; and the ability to provide clear technical information. For example, interviewees explained how messages trying to convey essential information could easily be lost if language was too technical. One of the interviewees reported using non-technical language to convey important safety messages to a local community in West Africa:

*“And then information, for example, if you give someone who is not a health professional and tell them malaria is transmitted like this, you need to put a mosquito net in your hotel. They appreciate that. Just tell them it is dangerous, and it can kill, how can you prevent it. Did you understand?”*

#### Relationship Building

Relationship building was coded as a non-technical skill required for effective response. Behaviors that helped build relationships included; informal communication channels; developing and maintaining trust; and establishing inter-organizational connections. Participants noted that the ability to build personal relationships was particularly important. Where new networks were created this was done through the development of personal relationships rather than formal approaches through organizations “*I think that personal relationships and informal channels are key”.* Participants also reported using existing networks, drawing information from a range of partner organizations. These networks provided detailed information that aided decision-making:

*“I met with every person I could identify in professional settings and communities, I collected advice from essentially field colleagues in all agencies and organization, health care workers, spiritual and political leaders, officials, schoolteacher, patients, families, young and older, survivors and parents of deceased, etc. I listened for 2 weeks before making formal proposals taking decisions.”*

Several participants also reflected on the importance of relationship building between responders and members of the community. One of the participants reported being asked to represent the community at a meeting with government officials, another that they were part of celebrations when Ebola patients returned to their communities:

*“You were part the team which went in and convinced them (the local population infected with Ebola) to get out from their homes. And we told them we would look after you when he came back. You have to go back and grieve and tell them we are sorry. And if they recover you become part of the celebration.”*

#### Coordination

Public Health responders noted that coordination was a key non-technical skill required to enact group decisions. Effective coordination was supported by the ability of team members to collaborate; understand the roles, responsibilities and capacities of stakeholders and adapt ways of working. Participants described how tasks were allocated according to pre-defined specialisms and roles, or dependent on the geographical areas of operation in which they were working.

*“we needed to work out how we worked …, contact tracing was UNFPA, risk communication was UNICEF, and there’s co-partners, there’s WHO, there’s [Ministry of Health], MSF with IMC, there’s so many people and logistics which the military take… once we divided that, then during the meetings everybody used to use it …”*

*“[you must work out] how can you maximize the efficiency gains you have from your team to work on the different elements of the response and that means re-purposing some staff members from what they were doing something else.”*

#### Decision-Making

The selection of a strategy or course of action in the MTS combined several different elements that were necessary to enable participants to manage the decision-making process. This included both individual decision-making and joint decision-making. Joint decision-making relied on the ability to incorporate a wide range of perspectives from across different organizations to reach consensus on a strategy and the ability to decide on a course of action while balancing immediate gain with longer term maintenance of the response:

“*yes very much so and that discussion went on quite a bit because based on people’s backgrounds and their training, and I don’t know maybe to some degree, their experience there were lots of different differing opinion, so I very much, we sort of had, in these situation a committee formed and we started to talk about what we were going to do.”*

Furthermore, participants reflected on the need to remain mindful of relationships with other agencies when making decisions that affected the wider network:

“*I was not happy with the quality of the plan… but it was it was a decision that I consciously made because not to jeopardize relationships… knowing that from a diplomatic or interpersonal relationship it’s better to be fairly flexible initially…”*

There were also indications that the networks used by responders provided an element of social support when making challenges decisions. Participants reflected that there was a sense of security when sharing the decision-making process, with one participant indicating that working as part of the group took the pressure out of the decision-making process.

*“I made the decision but after consulting with my colleagues. I relied heavily on my experience, but it was useful to have someone to bounce ideas off because I had no concrete idea of what to do.”*

#### Leadership

Participants described the importance of leadership in the multi-team environment to facilitate interoperable working across agencies. Participants described an absence of a clear command structure “*there was a bit of confusion as to who’s going to coordinate who’s going to do this and that”* and instances where organizations remained protective of their independence. This created difficulties, with participants reliant on other non-technical skills such as relationship building and communication to maintain the network and coordinate decision-making.

#### Personal Control and Experience

Participants highlighted that prior experience *“I had worked in this type of environment before and so I knew what had to be done from a public health perspective”* and the ability to maintain personal control over their emotions were important non-technical skills during the response to major disease outbreaks. For instance, some participants reflected on the need to return to protocols or core beliefs to help guide their decision-making, citing resilience, self-awareness, and humility as critical aspects of maintaining a level of personal control in changing circumstances. Notably several participants discussed the need to remain calm during periods of intense pressure and continuing to remind mindful of the longer-term outcomes of their efforts. Their prior experience responding in stressful environments and an ongoing desire to fulfill their objectives provided them with the ability to manage the pressure effectively:

“*I’ll talk about the pressure on a personal level, the pressure on a personal level was, I wanted to succeed. I also didn’t want [us] to be the last district to still be seeing cases.” That was one pressure, I had to work extra hard to ensure cases, are going down, we are moving, we are pushing the team.*

#### Situation Awareness

Participants reported that the ability to maintain situation awareness was an important non-technical skill within the wider multi-team environment. Participants discussed the need to obtain (and synthesize) information from a number of different sources in order to establish a full understanding of the response:

“*We needed to understand why the communities were hostile to response teams and what would happen if we stopped working. I also engaged with the UN peacekeepers who were providing security to take advice from them. So, I was using a combination of qualitative and quantitative data.”*

Further, participants suggested that a key element of establishing situation awareness and sensemaking was to remain mindful of the motivations of other actors within the complex system. Participants reported that different teams within an organization or structure can bring with them distinct perspectives which means that they can view information in a different way and apply different values to it. This difference between viewpoints or understanding (a representational gap) led to what some of the participants referred to as “silo working” where information from one component team could be ignored by others. Alternatively, participants also discussed the difficulties that arose when information was not shared effectively across different teams, leading to a failure in situation awareness and a lack of understanding:

*“the epidemiologists, the anthropologists, they had information, but they were keeping it. And this information was very, very crucial. For example, anthropologists are people who go to the ground and figure out why are people running away from the treatment center. Do you understand?”*

## Discussion

This study indicates that the emergency response environment in which public health professionals respond fits the definition of a complex MTS. The insights and descriptions provided by the public health professionals are consistent with the definition of MTSs and spoke to the difficulties of coordinating activities across component teams, each with different priorities and agendas (Marks et al., 2001; Zaccaro et al., 2012; Luciano et al., 2018). Furthermore, participant also reflected on the dynamic and fluid nature of the response environment, consistent with the view that MTSs can be characterized by their *ad hoc* nature, with teams joining and leaving the response dependent on the demands in the environment (Luciano et al., 2018; Brown et al., 2021). Participants reflected closely on the fact that in this environment work is undertaken not only through formed teams but also through formal and informal networks which are created and leveraged to gather and share information; to pool resources and expertise to respond effectively to the emergency. As the needs of the response change, networks that span different teams or organizations will change according to the task that needs to be performed.

A strong degree of overlap was identified here in the NTSs utilised by public health professionals and those identified by Flin et al. (2008). Core skills identified included—*situation awareness, communication, coordination, personal control and experience, relationship building, leadership* and *decision-making*. Interestingly, the context in which NTSs were discussed in the current study were largely relationship focused as opposed to task focused. This is somewhat at odds with Flin’s definition of NTSs being used as means to “contribute to safe and efficient task performance” (Flin et al., 2008). This focus on relationship building appeared to impact the way in which individuals engaged in NTS in a multi-team environment, and speaks to the primary challenge of operating within an MTS—to manage and coordinate activities across different component teams.

For instance, *situation awareness, decision-making, leadership and coordination* become group activities directed not only to the completion of tasks but also toward the development and maintenance of the networks necessary to complete those tasks. This finding indicates that we should view the response system not only in terms of boundary spanning between teams (see Carter, 2014), but as an ecosystem—where networks link teams and members together to develop situation awareness, take decisions and collaborate to take action. Conceptually this finding has interesting implications for the broader MTS literature, in which a focus on task completion has largely been used in which to frame and understand the behaviors of individuals operating within the system (see Firth et al., 2015; Brown et al., 2021). We suggest that future work on MTS may pay closer attention to the way in which relationships across component teams emerge and evolve over time and how the behaviors of individuals within the network support the development of a tightly linked eco-system.

### Practical Implications

The findings of this research have important implications for future practice. Here we highlight key take-aways that have the potential to support responders in a public health emergency.

• *Inter-team coordination is conducted using networks that are created and maintained during an emergency.*
  Responders can learn to identify and leverage potential and existing networks that exist in the response environment. Research can be carried out to examine methods for the identification of both informal and formal networks that are part of the multiteam system (response eco-system). Further research is required to examine how to leverage existing response systems and mechanisms to recognize, utilize, promote and sustain formal and informal networks.
• *Staff working in MTSs use NTS to build relationships between individuals and teams to create and maintain networks.*
  The value of relationship building can be encouraged in response training and the development of networks encouraged through the establishment of communities of practice. Sharing of information was identified as a key element to helping to build and maintain trust between teams in the MTS, consistent with prior research (Waring et al., 2020; Brown et al., 2021). The importance and role of individual relationship (as advocated by Janssen, 2010; Isabelle et al., 2012) building in addition to liaison between teams was emphasized by participants. The value of building individual relationships across the response can be recognized in doctrine and reinforced in training whilst methods of building individual relations between individuals working in distinct teams be examined.
• *In addition to “formal” information gathering and analysis systems responders use “informal” networks to gather information and check their understanding of the situation.*
  Responders should examine how to coordinate and utilize information gathered from formal and informal sources to inform situation analysis and influence decision-making. This can include but should not be limited to social media and contact with communities and individuals close to the response in addition to sources already used in disease and health systems surveillance (see Reuter and Kaufhold, 2018). Training for information managers and team leaders should include the importance of “systems thinking” and the development of a holistic response picture.
• *People working in MTS engaged in joint leadership and decision-making*.
  The need to recognize the interests and requirements of all parties has been documented in the literature on MTS. Some studies have viewed the emergence of a “dominant” team within the MTS as counter-productive to team performance (Brown et al., 2021). Further research is required into the nature of joint leadership and the concept of “enabling leadership.” Training can emphasize the importance of joint or shared leadership and the importance of recognizing the interests of other teams in the MTS and leveraging those to share resources including expertise. In the MTS, leadership is “as much influenced by an individual’s effectiveness in working in networks as it is by their narrower hierarchical parameters” (Kapacu and van Wart, 2008, p. 714). Linked to the concept of joint leadership is that of group decision-making which is common in humanitarian settings (ALNAP, 2016; Comes, 2016; Baharmand et al., 2020) although it is not clear from this research to what extent the group was used to identify options in the decision-making process, it was shown that the group was used to validate the decision that was made. Further research is required into the process of decision-making as a group whilst training should emphasize not only the value of group decision-making in accessing expertise but the role of joint decision-making in building trust between teams and individuals in the MTS.

### Limitations and Future Directions

Our findings concur with the view that in complex emergency environments, technical knowledge and systems alone are not enough. Whilst we recognize that our study has limitations, our findings concur with the recommendation that response systems must allow for the interaction between static procedures and a human-being’s capacity to learn, innovate and adapt to changing circumstances (Comfort et al., 2004; Comfort and Kapacu, 2006; Comfort, 2007). A research agenda which supports the elicitation, sharing and translation of expert knowledge about MTSs in this field, alongside the acquisition and development of non-technical skills is ripe for investigation (Ward et al., 2020).

Specifically, research might examine how to expedite communication across multi-team partners, to develop networks and personal links in the preparedness as well as response phases (Moshtari, 2016; Waring et al., 2020). Communication was identified as key here in enabling teams to enact joint decisions, achieve shared awareness and to establish trusting relationships over time. Future research may also explore whether, how and indeed when shared leadership could be adopted across the MTS and how best to allocate responsibilities in roles. Including how people navigate the bridge between networks (both formal and informal) and response structures such as the *Incident Management System and Cluster system.* Scholars including Andreassen et al. (2020) argue that a series of networks within the response environment provide a means for the looser coordination that supports the formal support structures.

To support this kind of research, researchers will need to think creatively about how to develop after action review methods and immersive simulation scenarios that will elicit the professional non-technical skill requirements identified here (*see* Brown et al., 2020).

Indeed, as recently noted by Fischer and Mosier (2020), the small sample sizes and qualitative inquiry that has characterized research into MTS has limited the possibility for statistical analysis. The cross-sectional nature of this study makes it difficult to attribute causality and can lead to bias (Sedgewick, 2014; Taris et al., 2021). The potential for bias is also present in the choice of interview as a method (Schwarz and Oyserman, 2001; Alsaawi, 2014). The issues were mitigated as far as possible in the study design by piloting the interviews; by conducting semi-structured interviews that allowed dialogue for clarification and repetition of the narrative; by providing background information and prompts and by emphasizing the importance of the experience of the participant without any judgment, reiterating that decisions made in the response environment are often imperfect (Klein et al., 2010). Moving forward, we hope, like them, that collecting data across simulations and sharing data among researchers will increase—“researchers must be as collaborative as the MTS teams they wish to understand” (Fischer and Mosier, 2020:846).

## Conclusion

In concluding, we note that the complexity of MTS, as the Covid-19 pandemic continues to show, is constantly challenged by temporal considerations and increasingly by adaptive technology and considerations of scale. The challenge as we have shown however, continues to be at the human level, and a focus upon non-technical skills provides insight into behaviors and to some extent cognitions, which were previously not examined in the case of public health professionals at a meso level of analysis. Our findings also suggest that Public Health professionals must take a holistic view of the response—including knowledge of the socio-economic and political context but also of response mechanisms and the relationships between different organizations and individuals. Further study of the interconnectedness of MTSs, and how responders use non-technical skills in complex environments will add to both practitioner and academic collaboration, understanding and elicitation. The more we can explore and understand the behavior of the professionals working at the hard end of MTS, the more we can advance both science and practice and our understanding of paradoxes and practicalities.

## Data Availability Statement

The datasets presented in this article are not readily available because the data is not publicly available. Requests to access the datasets should be directed to AB, andrewblack@who.int.

## Ethics Statement

The studies involving human participants were reviewed and approved by University of Bath, United Kingdom. The participants provided their written informed consent to participate in this study.

## Author Contributions

AB: writing and data collection/analysis. OB: editing and writing. HU and GG: methods design. JG: writing, drafting, and editing—coordinating academic/practitioner collaboration. All authors contributed to the article and approved the submitted version.

## Conflict of Interest

The authors declare that the research was conducted in the absence of any commercial or financial relationships that could be construed as a potential conflict of interest.

## Publisher’s Note

All claims expressed in this article are solely those of the authors and do not necessarily represent those of their affiliated organizations, or those of the publisher, the editors and the reviewers. Any product that may be evaluated in this article, or claim that may be made by its manufacturer, is not guaranteed or endorsed by the publisher.
